# Gene Expression Analysis of Three Putative Copper-Transporting ATPases in Copper-Tolerant *Fibroporia radiculosa*

**DOI:** 10.3389/fmicb.2020.586940

**Published:** 2020-12-04

**Authors:** Katie M. Ohno, Amy B. Bishell, Glen R. Stanosz

**Affiliations:** ^1^USDA Forest Service, Forest Products Laboratory, Madison, WI, United States; ^2^Department of Forestry and Wildlife Ecology, University of Wisconsin-Madison, Madison, WI, United States

**Keywords:** copper-tolerance, brown-rot fungi, wood decay, gene expression, ATPases

## Abstract

Copper tolerance of brown-rot basidiomycete decay fungi can lessen the efficacy of copper-containing wood preservatives for wood products in-service. The purpose of this study was to evaluate wood mass loss and differential expression of three genes that have putative annotations for copper-transporting ATPase pumps (FIBRA_00974, FIBRA_04716, and FIBRA_01430). Untreated southern pine (SP) and SP treated with three concentrations of ammoniacal copper citrate (CC, 0.6, 1.2, and 2.4%) were exposed to two copper-tolerant *Fibroporia radiculosa* isolates (FP-90848-T and L-9414-SP) and copper-sensitive *Gloeophyllum trabeum* isolate (MAD 617) in a 4-week-long standard decay test (AWPA E10-19). Decay of copper-treated wood was inhibited by *G. trabeum* (*p* = 0.001); however, there was no inhibition of decay with increasing copper concentrations by both *F. radiculosa* isolates. Initially, *G. trabeum* and one *F. radiculosa* isolate (L-9414-SP) highly upregulated FIBRA_00974 and FIBRA_04716 on copper-treated wood at week 1 (*p* = 0.005), but subsequent expression was either not detected or was similar to expression on untreated wood (*p* = 0.471). The other *F. radiculosa* isolate (FP-90848-T) downregulated FIBRA_00974 (*p* = 0.301) and FIBRA_04716 (*p* = 0.004) on copper-treated wood. FIBRA_01430 expression by *G. trabeum* was not detected, but was upregulated by both *F. radiculosa* FP-90848-T (*p* = 0.481) and L-9414-SP (*p* = 0.392). Results from this study suggest that all three test fungi utilized different mechanisms when decaying copper-treated wood. Additionally, results from this study do not provide support for the involvement of these putative gene annotations for copper-transporting ATPase pumps in the mechanism of copper-tolerance.

## Introduction

Copper is an essential metal for eukaryotic life (Lattore et al., 2011; Smith et al., 2017; Raffa et al., 2019). It functions in generating energy, mobilizing iron, inactivating enzymes, interacting and stabilizing ligands, and generating and detoxifying reactive oxygen species (ROS; Li et al., 2005; Smith et al., 2017; Raffa et al., 2019). Fungal cells uptake copper through ion exchange which permeates copper throughout and leads to accumulation inside the cell (Somers, 1963). Affinity, distribution, and accumulation of copper vary among fungal species. Fungi must strictly regulate copper levels as excess can cause oxidative damage, enzyme inhibition, interruptions in nutrient transport, protein and enzyme denaturation, or cellular death (Freeman and McIntyre, 2008; Smith et al., 2017).

Fungi utilize several mechanisms to deal with toxic metals in their immediate environment (Gadd, 1993; Hall, 2002). These metal tolerance mechanisms of fungi appear to include: efflux and excretion of accumulated metals (Weissman et al., 2000; Ito et al., 2007); cell wall complexation facilitated by extracellular polymeric slime materials which aid in controlling metal uptake (Daniel and Nilsson, 1989; Yahaya et al., 2009); chelation and/or precipitation by soluble metabolites (oxalic acid; Clausen, 2000; Clausen and Green, 2003; Green and Clausen, 2003; Jarosz-Wilkolazka and Graz, 2006; Tang et al., 2013); accumulated metal complexation and immobilization by metallothioneins (Schwartz et al., 2013); and metal sequestration to the periplasm or vacuoles (Pearce and Sherman, 1999). Each of these mechanisms can be employed singularly or in concert to prevent metal toxicity by reducing intracellular damage typically caused by oxidative species resulting from reactive metal ions.

Some decay fungi are able to successfully colonize and destroy copper-treated wood (DeGroot and Woodward, 1999; Hall, 2002; Green and Clausen, 2003). Copper-tolerant fungi thrive in the toxic environment of treated wood due to their ability to render copper ions found in treated wood inert (Gadd, 1993; Hastrup et al., 2005). Many copper-tolerant fungi produce high amounts of oxalic acid which precipitates copper ions as insoluble copper-oxalate and has been proposed to explain how these copper-tolerant fungal organisms overcome unfavorable copper environments (Shimanzono and Takubo, 1952; DeGroot and Woodward, 1999; Pohleven et al., 1999; Humar et al., 2002; Green and Clausen, 2003, 2005; Hastrup et al., 2005, 2006; Freeman and McIntyre, 2008; Arango et al., 2009; Schilling and Inda, 2011; Tang et al., 2013). It is hypothesized that high extracellular oxalic acid accumulation aids in the precipitation of copper ions into insoluble copper oxalate crystals, inactivating copper in this process (Gadd, 1993; DeGroot and Woodward, 1999; Green and Clausen, 2003; Hastrup et al., 2005; Freeman and McIntyre, 2008). Additionally, it has been documented that copper-tolerant fungi produce two to 17 times more oxalic acid when exposed to copper-treated wood compared to non-treated wood (Green and Clausen, 2005; Jenkins, 2012; Ohno et al., 2015).

Several studies including *Postia placenta*, *Antrodia vaillantii*, *Fomitopsis (Tyromyces) palustris*, *Meruliporia (Poria) incrassata*, *Wolfoporia cocos*, and *F. radiculosa* revealed the presence of copper-oxalate moolooite crystals produced on copper-treated wood (Murphy and Levy, 1983; Sutter et al., 1983, 1984; Tsunoda et al., 1997; DeGroot and Woodward, 1999; Clausen et al., 2000; Tang et al., 2013). Subsequent gene expression analysis showed that increased oxalate production in the presence of a copper-based preservative was regulated by increased expression of key genes in glyoxylate ametabolism (Tang et al., 2013). Hence, it has become generally accepted that oxalic acid is integral to the mechanism of copper-tolerance (Murphy and Levy, 1983; Sutter et al., 1983; Daniel, 1994; Leithoff et al., 1995; Pohleven et al., 2002; Green and Clausen, 2003, 2005; Hastrup et al., 2006; Freeman and McIntyre, 2008; Arango et al., 2009; Schilling and Inda, 2011; Tang et al., 2013). However, direct availability of oxalic acid, its accumulation, or copper oxalate precipitation does not always correlate with increased mass loss in copper-treated wood (Sutter et al., 1983; DeGroot and Woodward, 1999; Clausen, 2000; Schilling and Jellison, 2005; Ohno et al., 2017). Additionally, the effect of sodium oxalate added to non-treated and copper-treated wood does not increase mass loss caused by copper-tolerant fungi; therefore, it was suggested that oxalic acid accumulation and precipitation are not the exclusive method copper-tolerant fungi utilize in overcoming high copper concentrations (Schilling and Jellison, 2005; Ohno et al., 2017).

Copper-transporting ATPases are evolutionarily conserved proteins that mediate import, distribution, sequestration, and export of cellular copper (Migocka, 2015). In eukaryotes, ATPases are found in intracellular compartments, the Golgi membrane or the plasma membrane (depending on their primary function) serve in metal detoxification and shuttles copper to copper dependent enzymes in the secretory pathway (Gupta and Lutsenko, 2012). In yeast, copper homeostasis is not universal and specific proteins differ among genera. In *Saccharomyces cerevisiae*, high intracellular copper levels are sensed by transcription factor *Ace1*, which activates metallothioneins, *Cup1* and *Crs5*, and a superoxide dismutase (*Sod1*; Raffa et al., 2019). In *Cryptococcus neoformans*, high intracellular copper levels are sensed by *Cuf1*, which activates metallothioneins, *Cmt1* and *Cmt2* to bind excess copper (Raffa et al., 2019). In *Candida albicans*, high intracellular copper levels are sensed by *Cup2*, which activates a copper exporter, *Crp1*, and metallothioneins *Cup1* and *Crd2* remove or bind excess copper (Raffa et al., 2019). In *C. albicans*, copper-transporting ATPase pumps are responsible for preventing intracellular copper concentrations from becoming toxic (Weissman et al., 2000).

In *Fibroporia radiculosa* (Peck) Glib & Ryvarden, three genes with function related to regulating copper concentrations were detected (Tang et al., 2012). Tang et al. (2012) determined that of the three putative gene annotations for ATPases, only one (FIBRA_00974) encoded a signal peptide and another (FIBRA_01430) had no homolog in copper-tolerant brown-rot decay fungus *Serpula lacrymans*, which could be indicative of the higher tolerance observed in *F. radiculosa*. Data for involvement of these genes in regulating copper tolerance, however, were conflicting (Tang et al., 2013). Although transcriptomics analysis suggested involvement of one COP-encoding gene (FIBRA_01430), the follow-up experiment using qRT-PCR showed this gene was not upregulated during decay of wood treated with a copper-based preservative compared to untreated wood (Tang et al., 2013). Jenkins (2012) measured expression of FIBRA_01430 in four isolates of *F. radiculosa* on copper-treated wood over an 8 week period and found expression was greater in the presence of copper. Based on these findings, it was hypothesized that this ATPase gene functions to prevent accumulation of toxic concentrations of copper in the cell during the early stages, and that this is a necessary step in initial colonization of copper-treated wood by *F. radiculosa* (Jenkins, 2012).

The goal of this study was to expand our current understanding of the genes thought to be involved in copper-tolerance of *F. radiculosa* during initial decay. We examined the differential expression of three putative copper transporting ATPase genes (FIBRA_00974, FIBRA_04716, and FIBRA_01430) in two copper-tolerant *F. radiculosa* isolates (FP-90848-T and L-9414-SP) and copper-sensitive *Gloeophyllum trabeum* (pers. Ex Fr.) Murr. (MAD 617) during decay of untreated and copper-treated SP wood. Implications from this study could specify genes responsible for overcoming unfavorable copper environments in copper-tolerant decay fungi.

## Materials and Methods

### Fungal Cultures

Two copper-tolerant *F. radiculosa* isolates (FP-98048-T and L-9414-SP; USDA-NRS-FMHC, Forest Products Laboratory, Madison, WI, United States) were used. One copper-sensitive *G. trabeum* (MAD 617; USDA-NRS-FMHC, Forest Products Laboratory, Madison, WI, United States) was also used. Fungal cultures were maintained on malt extract agar (BD, ThermoFisher Scientific, Waltham, MA, United States) at 27°C and 70% relative humidity (RH).

### Preservative Treatment and Decay Tests

Experimental set-up consisted of accelerated decay tests following the American Wood Protection Association Standard E10-19 (American Wood Protection Association, 2019). SP wood wafers (40 by 30 by 3 mm) in soil block jars (one wafer/jar) were initially colonized for 3 weeks after inoculation by fungal mycelial plugs. SP test blocks (20 mm cubes) were vacuum-treated with three concentrations of ammoniacal copper citrate: 0.6% (3.350 kg Cu/m^3^), 1.2% (6.888 kg Cu/m^3^), and 2.4% (13.642 kg Cu/m^3^). Untreated SP blocks were included. After treatment, blocks dried under a hood overnight and were conditioned to 27°C and 30% RH for 2 weeks. Wood block exposure to the fungi was accomplished by aseptically placing two test blocks (steam sterilized 20 min, 122°C and cooled to room temperature) onto the colonized wood wafer in each glass bottle for each fungus and time point. Bottles were then incubated at 27°C and 70% RH. Weekly for each of 4 weeks, one test block from each of three glass bottles for each treatment was used to measure mass loss (*n* = 3). Test blocks were lightly brushed free of mycelia, oven-dried overnight (60°C), and reconditioned (27°C and 30% RH) for 2 weeks prior to weighing and calculating mass loss (%). The second test block from the same jar was used for RNA extractions (*n* = 3).

### Sample Preparation and RNA Extraction

Approximately, 0.2 g of sawdust was produced by drilling into each test block with a sterile bit and placed into individual microtubes, and RNA was immediately extracted. The Ambion® RNAqueous™ Kit (ThermoFisher Scientific) was used to isolate RNA following the manufacturer’s specifications with an added DNaseI (Promega Corporation, Madison, WI, United States) digestion to removed unwanted genomic DNA contamination. DNaseI digestion was prepared according to the manufacturer’s specifications and was included between the Wash Solution 1 step and Wash Solution 2 step of the RNAqueous™ Kit. RNA for all samples was quantified by a NanoDrop 2000 spectrophotometer (ThermoFisher Scientific). Yields ranged from 2 to 12 ng g^−1^ sawdust. RNA samples were stored at −80°C.

### Protein Extraction and Western Blot

Fungal hyphae of the two *F. radiculosa* isolates were grown in 50 ml Bailey’s minimal media with 1% cellobiose and 2.5% glucose for 9 days at 27°C and 70% RH (Highley, 1973). Three replicate cultures were grown in untreated Baileys minimal media, Bailey’s minimal media with 3 mM copper sulfate, and Bailey’s minimal media with 1 mM sodium orthovanadate, an ATPase inhibitor. Whole protein extractions were conducted using a modified version of Zhang et al. (2011). Fungal hyphae (0.1 g) were collected by vacuum filtration and then homogenized in 1 ml of 2 M lithium acetate using an Omni probe homogenizer (3 × 5 s pulses; Omni International, Kennesaw, GA, United States). Homogenate was held at room temperature for 10 min and then centrifuged at 4,000 × *g* for 5 min at 4°C. The pellet was resuspended in 1 ml of 0.4 M sodium hydroxide by vortexing 5 s and then held on ice for 5 min. The homogenate was then centrifuged 10,000 × *g* for 5 min at 4°C. The pellet was resuspended in SDS buffer (1% SDS, 100 mM Tris-HCL pH 7, 1 mM protease inhibitor), by mixing with the pipet tip and then boiled 5 min at 100°C. Finally, this homogenate was centrifuged 4,000 × *g* for 5 min at room temperature and pellet was discarded. Protein fractions were quantified using the *RC DC*™ Protein Assay (Bio-Rad Laboratories) following the manufacturer’s specifications.

Protein fractions (0.5–5 μg) were loaded onto a NuPAGE™ 4–12% Bis-Tris polyacrylamide gel (ThermoFisher Scientific) and run in NuPAGE™ MOPS SDS buffer (ThermoFisher Scientific) at 100 V for 50 min. Resolved proteins were transferred to a nitrocellulose membrane (Bio-Rad Laboratories) in NuPAGE™ Transfer buffer (ThermoFisher Scientific) at 30 V for 60 min. The membrane was blocked with 3% Blotting Grade Blocker (Bio-Rad Laboratories) for 60 min and probed with ATP7A raised toward a human copper-translocating P-type ATPase protein (Aviva Systems Biology, San Diego, CA, United States) at a 1:500 dilution overnight. Secondary probing with Goat Anti-Rabbit IgG (H + L)-HRP conjugate (Bio-Rad Laboratories) at a 1:3000 dilution for 60 min followed. Blots were developed using the Immun-Blot® Opti-4CN Colorimetric Goat-anti-Rabbit kit (Bio-Rad Laboratories) following the manufacturer’s specifications and visualized on a Gel Doc XR+ system (Bio-Rad Laboratories). A positive control (EGY48 yeast lysate) for ATP7A was included (Santa Cruz Biotechnology, Inc., Dallas, TX, United States).

### First Strand cDNA Synthesis

RNA (20 ng per 20 μl reaction mixture) was synthesized to first strand complementary DNA (cDNA) using the SuperScript™ II Reverse Transcriptase Kit (Invitrogen, Carlsbad, CA, United States) following the manufacturer’s specifications. cDNA synthesis was carried out on an MJ Research PTC-225 Thermal Cycler (Bio-Rad Laboratories, Carlsbad, CA, United States) with the following settings: incubation at 42°C for 50 min and inactivation at 70°C for 15 min. Both non-template controls and reverse transcriptase free controls were included. cDNA samples were stored at −20°C.

### qPCR Analysis

Gene-specific primers were designed using gene and coding sequences for FIBRA_00974, FIBRA_04716, and FIBRA_01430 in *F. radiculosa* through NCBI GenBank. For the transcripts, primer pairs spanned at least one intron. Amplicon lengths ranged from 299 to 900 bp depending on the gene (FIBRA_00974: 649 bp; FIBRA_04716: 299 bp; and FIBRA_01430: 900 bp). Table 1 lists the primer sequences for the target genes and endogenous control. A preliminary electrophoresis analysis was performed from RT-PCR products for each gene and non-template and reverse-transcriptase-free controls on a 2% agarose gel in TAE (1x) buffer. Target bands were verified based on amplicon length for the respective genes. Additionally, target bands were absent from controls but present in samples for the respective amplicon lengths.

**Table 1:** Primer sequences for FIBRA_00974, FIBRA_04716, and FIBRA_01430 genes and *β*-actin (housekeeping gene) designed for this study.

Target gene	Primer sequence
FIBRA_00974_F	5'-TCACTTCTCACAACCTCGTTACT-3'
FIBRA_00974_R	5'-CCCAGCATCGTTAGCACATA-3'

FIBRA_04716_F	5'-CGGTCTTATCGAGTGCCTTTAG −3'
FIBRA_04716_R	5'-GGCATCAAAGCCCATTTCTTC-3'

FIBRA_01430_F	5'-TTGAGGACAGCAAGGGAAAG-3'
FIBRA_01430_R	5'-CTGATGATGAACGTCGGGATAG-3'

β-actin_F	5'-GTGATGGTTGGTATGGGTCAGAAGG-3'
β-actin _R	5'-GAAGCTCGTTGTAGAAAGTGTGATGC-3'

Expression profiles of FIBRA_00974, FIBRA_04716, and FIBRA_01430 were monitored in real time using a MyiQ™ 5 Real-Time Detection System (Bio-Rad Laboratories). Sample preparations (undiluted cDNA) were carried out using the iQ™ SYBR® Green Supermix (Bio-Rad Laboratories) following the manufacturer’s specifications. Three biological replicates were run in duplicate for each plate against the non-template and reverse transcriptase free controls for each of the treatments, time points, and genes. The reaction protocol included an initial denaturation at 95°C for 3 min, followed by 40 cycles of a 15 s 95°C denaturation, 30 s annealing at 60°C, and a 30 s 72°C extension. For FIBRA_01430, annealing was carried out at 55°C. A melt curve analysis of the resulting PCR product was performed immediately following amplification to check for primer specificity and primer dimer formation (1 min incubation at 95°C, followed by a 1 min annealing at 55°C prior to 81 cycles of 10 s with temperatures increasing by 0.5°C each cycle).

Using the housekeeping gene (*β*-actin; 270 bp amplicon) mean C_T_ values, average replicate C_T_ values were normalized for all plates. Normalized C_T_ values were used to calculate mean fold change (2^-*Δ*ΔCT^) in expression of the three ATPase genes using the comparative C_T_ method (Livak and Schmittgen, 2001). For the three test fungi, untreated wood at week 1 was used as the endogenous control for fold change calculations for FIBRA_00974, FIBRA_04716, and FIBRA_01430. Mean fold changes were then converted to the log_2_ scale to equally represent upregulation (values > 0) and downregulation (values < 0). Values reflect the differences between the endogenous control and each treatment in the subsequent weeks. If no value is shown in the subsequent figures, the gene was not expressed for that particular test variable.

### Statistical Analysis

A three-way ANOVA was performed using the general linear model (GLM) to determine effects of fungi, treatment, and time on mass loss. GLM describes statistical relationships between multiple factors using mean values to find significant differences. Mass loss data were reported in percentages, therefore, did not meet the assumptions of a parametric statistical analysis. Data were analyzed after applying the arcsine of the square root transformations to proportions.

Non-parametric statistical analysis using the Kruskal-Wallis test (*p* = 0.05) was conducted to determine differences among treatment and time for FIBRA_00974, FIBRA_04716, and FIBRA_01430 expression, respectively (Goni et al., 2009). Treatments were grouped for each fungus prior to statistical analysis. Additionally, time points were grouped for each fungus prior to statistical analysis. Statistical analyses were performed using Minitab 17.2.1 (Minitab, LLC, State College, PA, United States).

## Results

### Decay

**Figure 1:**
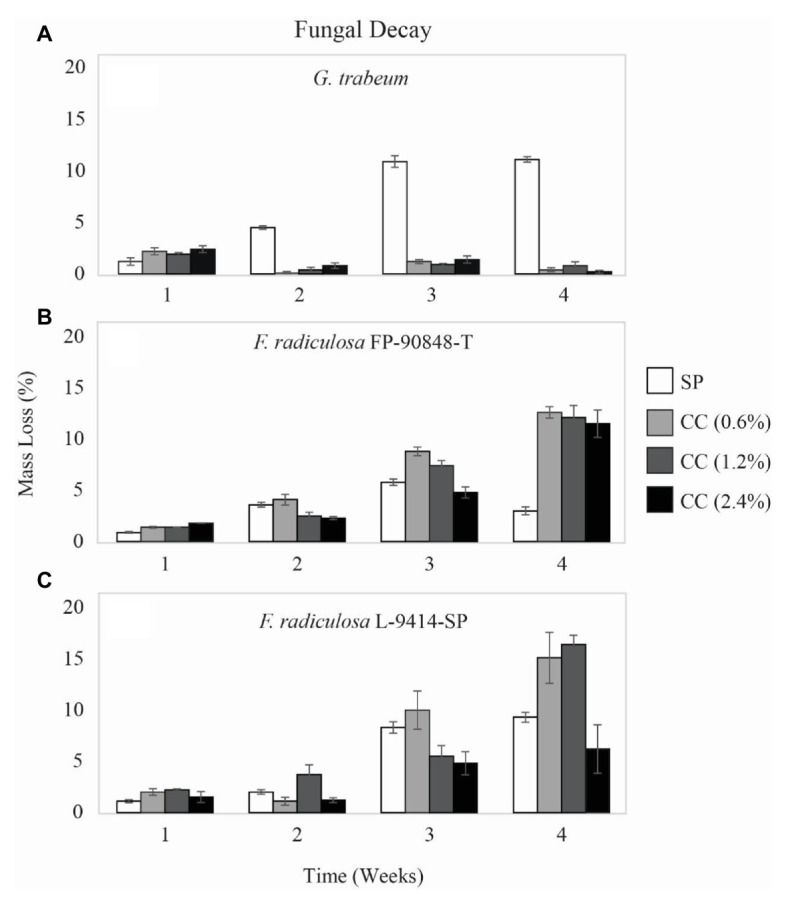
Mass loss (%) ± SE of untreated (SP), 0.6% ammoniacal copper citrate treated (0.6% CC), 1.2% ammoniacal copper citrate treated (1.2% CC), and 2.4% ammoniacal copper citrate treated (2.4% CC) southern pine (SP) test blocks exposed to: **(A)** copper-sensitive *Gloeophyllum trabeum* (MAD 617), **(B)** copper-tolerant *Fibroporia radiculosa* (FP-90848-T), and **(C)** copper-tolerant *Fibroporia radiculosa* (L-9414-SP) over the course of 4 weeks (*n* = 3).

Mass loss data (%) differed among the three test fungi and on untreated and treated wood over time (Figure 1). ANOVA showed significant effects of fungus, treatment, time, and all interactions (values of *p* ≤ 0.001). *Gloeophyllum trabeum* indicated copper sensitivity on copper-treated wood through 4 weeks (Figure 1A). On untreated wood, *G. trabeum* increased mass loss from 1.2% at week 1 to 11.1% at week 4. On copper-treated wood, *G. trabeum* produced significantly lower mass loss for all copper levels, < 2.4% (*p* = 0.001). In contrast, *F. radiculosa* FP-90848-T (Figure 1B) and L-9414-SP (Figure 1C) were not inhibited by copper-treated wood through 4 weeks. Mass losses caused by both *F. radiculosa* isolates on untreated wood were significantly lower (< 9%) than mass losses of copper-treated wood (*p* = 0.000). *Fibroporia radiculosa* FP-90848-T caused significantly greater mass losses on all concentrations of copper-treated wood compared to untreated wood by week 4, up to 12.6% (*p* = 0.001). *Fibroporia radiculosa* L-9414-SP caused significantly greater mass losses on all concentrations of copper-treated wood compared to untreated wood by week 4, up to 16.4% (*p* = 0.001). However, decay by *F. radiculosa* L-9414-SP was less on 2.4% CC by week 4 compared to FP-90848-T.

### Western Blot

**Figure 2:**
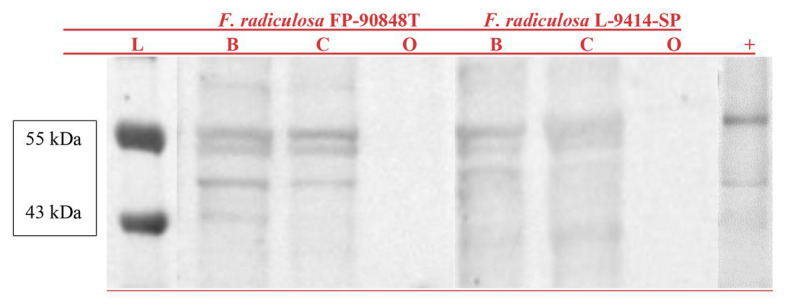
Western blot of ATP7A probed whole cell lysate protein fractions from *Fibroporia radiculosa* isolates FP-90848-T and L-9414-SP grown in Bailey’s minimal media (B) Bailey’s minimal media containing 3 mM copper sulfate (C), and Bailey’s minimal media with 1 mM sodium orthovanadate (O). A molecular marker (L) is represented on the left. An EGY48 yeast lysate with ATP7A reactivity is represented on the right (+).

Whole cell lysate protein fractions probed with an antibody raised to human ATP7A can be seen in Figure 2. Results show reactivity in both *F. radiculosa* isolates at 55 and 40 kDa when grown in Bailey’s minimal media and Bailey’s minimal media with 3 mM copper sulfate. Three bands were seen in both *F. radiculosa* isolates. Two bands at 55 kDa and a third band around 40 kDa. The three bands could correspond to the three putative copper-transporting ATPases characterized in *F. radiculosa*. There was no ATP7A reactivity when grown in Bailey’s minimal media with 1 mM of sodium orthovanadate, an inhibitor of ATPases, for both *F. radiculosa* isolates.

### FIBRA_00974

**Figure 3:**
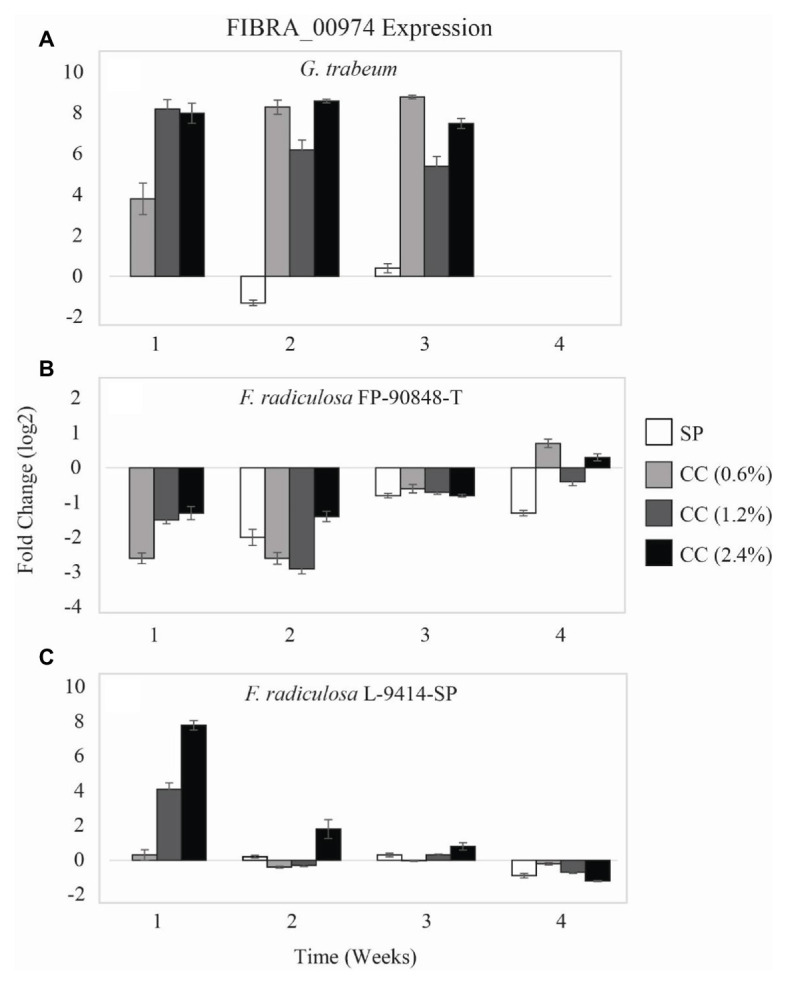
Gene expression values (log_2_) ± SE of the copper-transporting FIBRA_00974 gene of: **(A)** copper-sensitive *G. trabeum* (MAD 617), **(B)** copper-tolerant *F. radiculosa* (FP-90848-T), and **(C)** copper-tolerant *F. radiculosa* (L-9414-SP) on untreated (SP), 0.6% ammoniacal copper citrate treated (0.6% CC), 1.2% ammoniacal copper citrate treated (1.2% CC), and 2.4% ammoniacal copper citrate treated (2.4% CC) SP test blocks over the course of 4 weeks. Values above 0 indicate upregulation. Values below 0 indicate downregulation relative to expression on SP at week 1 for each fungus, respectively (set at 0). FIBRA_00974 was not expressed for *G. trabeum* at week 4.

Expression of a copper-transporting ATPase gene (FIBRA_00974) differed among the three test fungi exposed to untreated and copper-treated wood (Figure 3). *Gloeophyllum trabeum* (Figure 3A) upregulated FIBRA_00974 significantly more when exposed to copper-treated wood compared to untreated wood (*p* = 0.000). Additionally, there was no statistically significant difference of *G. trabeum* FIBRA_00974 expression for weeks 1–3 (*p* = 0.944). There was no detectable FIBRA_00974 expression for *G. trabeum* on untreated and copper-treated wood at week 4 despite housekeeping gene amplification.

The two *F. radiculosa* isolates differed in FIBRA_00974 expression over the course of this study. Generally, *F. radiculosa* FP-90848-T downregulated FIBRA_00974 on untreated and copper-treated wood (Figure 3B). However, there was minimal FIBRA_00974 upregulation when *F. radiculosa* FP-90848-T was exposed to 0.6 and 2.4% CC at week 4 compared to the other weeks. Additionally, there was no statistically significant difference of *F. radiculosa* FP-90848-T FIBRA_00974 expression between untreated and copper-treated wood (*p* = 0.301). *Fibroporia radiculosa* FP-90848-T downregulated FIBRA_00974 more at week 2 compared to the other weeks (*p* = 0.000). *Fibroporia radiculosa* L-9414-SP upregulated FIBRA_00974 more on copper-treated wood compared to untreated wood at week 1 (Figure 3C). Additionally, FIBRA_00974 upregulation increased with increasing copper concentration at week 1. However, the grouped analysis showed no differences in FIBRA_00974 expression for this isolate on untreated and copper-treated wood (*p* = 0.471). *Fibroporia radiculosa* L-9414-SP upregulated FIBRA_00974 more at week 1 compared to the other weeks and downregulated FIBRA_00974 more at week 4 compared to the other weeks (*p* = 0.000).

### FIBRA_04716

**Figure 4:**
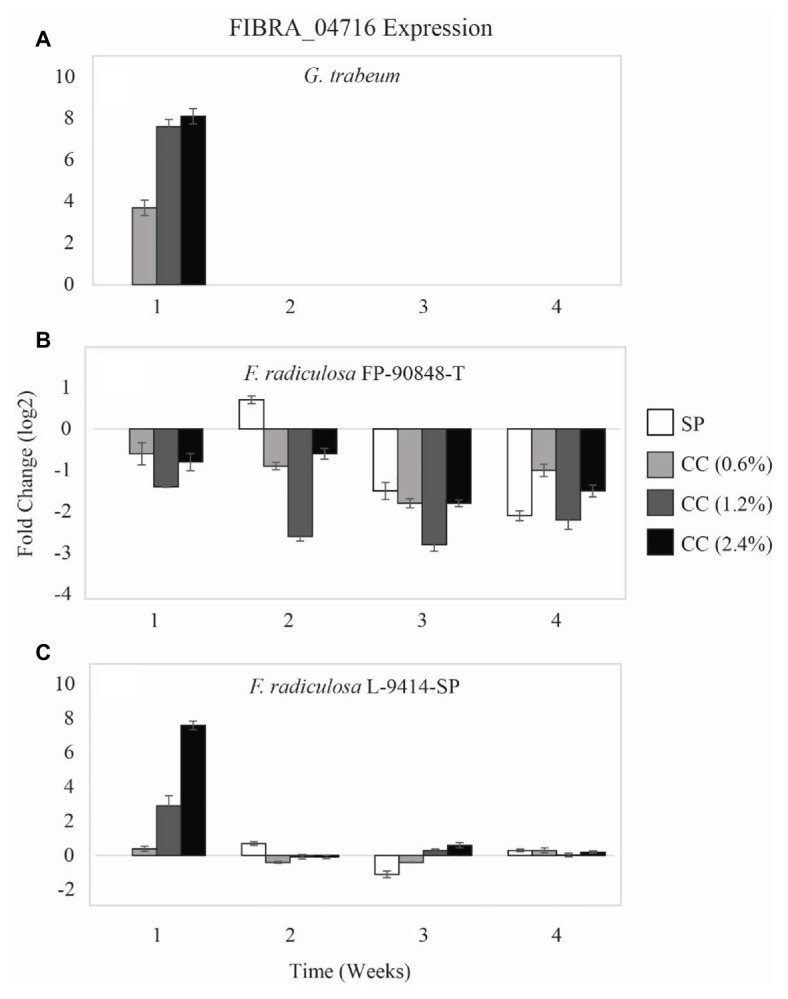
Gene expression values (log_2_) ± SE of the copper-transporting FIBRA_04716 gene of: **(A)** copper-sensitive *G. trabeum* (MAD 617), **(B)** copper-tolerant *F. radiculosa* (FP-90848-T), and **(C)** copper-tolerant *F. radiculosa* (L-9414-SP) on untreated (SP), 0.6% ammoniacal copper citrate treated (0.6% CC), 1.2% ammoniacal copper citrate treated (1.2% CC), and 2.4% ammoniacal copper citrate treated (2.4% CC) SP test blocks over the course of 4 weeks. Values above 0 indicate upregulation. Values below 0 indicate downregulation relative to expression on SP at week 1 for each fungus, respectively (set at 0). FIBRA_04716 was not expressed for *G. trabeum* at weeks 2–4.

A second copper-transporting ATPase gene (FIBRA_04716) differed in expression among the three test fungi on untreated and copper-treated wood (Figure 4). *Gloeophyllum trabeum* (Figure 4A) upregulated FIBRA_04716 more on copper-treated wood compared to untreated wood at week 1 (*p* = 0.005). After week 1, *G. trabeum* had no detectable FIBRA_04716 expression on untreated and copper-treated wood.

Again, the FIBRA_04716 expression patterns of the two *F. radiculosa* isolates differed over the course of this study. *Fibroporia radiculosa* FP-90848-T downregulated FIBRA_04716 (Figure 4B). This isolate had greater FIBRA_04716 downregulation on copper-treated wood compared to untreated wood at week 1 through week 3 (*p* = 0.004). However, by week 4, *F. radiculosa* FP-90848-T downregulated FIBRA_04716 more on untreated wood compared to copper-treated wood. Additionally, this isolate downregulated FIBRA_04716 more at week 3 compared to the other weeks (*p* = 0.001). Generally, *F. radiculosa* L-9414-SP showed FIBRA_04716 upregulation on untreated and copper-treated wood over the 4 weeks (Figure 4C); however, there were instances where FIBRA_04716 was downregulated (copper-treated wood, week 2). Also it should be mentioned at week 1, *F. radiculosa* L-9414-SP upregulated FIBRA_04716 more on copper-treated wood and increased upregulation with increasing copper concentration. These results are comparable to FIBRA_00974 expression at week 1 seen in this isolate, and to FIBRA_00974 and FIBRA_04716 expression results of *G. trabeum*. Despite having greater FIBRA_04716 upregulation at week 1 on copper-treated wood, the grouped analysis for this isolate on untreated and copper-treated wood lacked statistical significance in FIBRA_04716 expression (*p* = 0.059). *Fibroporia radiculosa* L-9414-SP upregulated FIBRA_04716 more at week 1 compared to the other weeks (*p* = 0.048).

### FIBRA_01430

**Figure 5:**
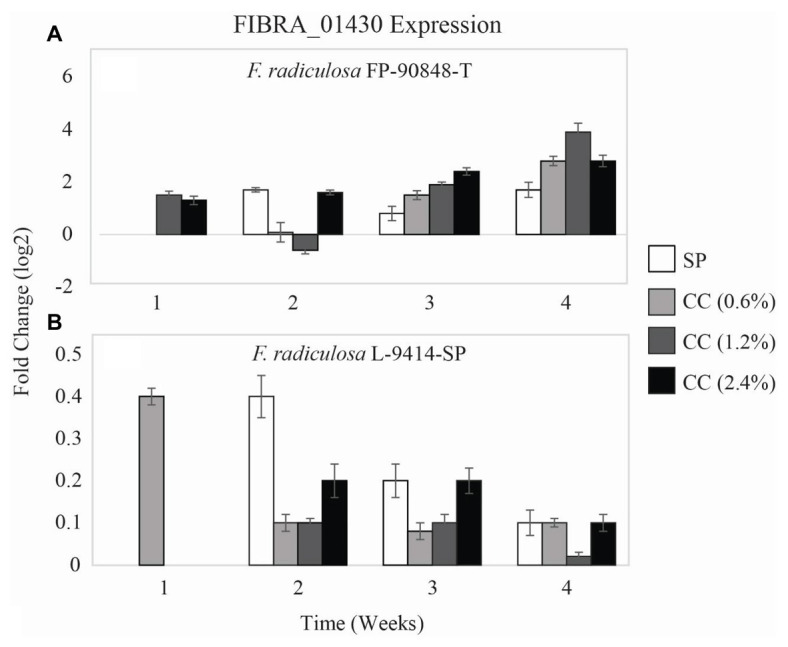
Gene expression values (log_2_) ± SE of the copper-transporting FIBRA_01430 gene of: **(A)** copper-tolerant *F. radiculosa* (FP-90848-T) and **(B)** copper-tolerant *F. radiculosa* (L-9414-SP) on untreated (SP), 0.6% ammoniacal copper citrate treated (0.6% CC), 1.2% ammoniacal copper citrate treated (1.2% CC), and 2.4% ammoniacal copper citrate treated (2.4% CC) SP test blocks over the course of 4 weeks. Values above 0 indicate upregulation. Values below 0 indicate downregulation relative to expression on SP at week 1 for each fungus, respectively (set at 0). FIBRA_01430 was not expressed for *G. trabeum* at weeks 1–4, *F. radiculosa* FP-90848-T on 0.6% CC at week 1, and *F. radiculosa* on 1.2 and 2.4% CC at week 1.

Two of the three test fungi differed in expression of a third copper-transporting ATPase gene (FIBRA_01430) on untreated and copper-treated wood (Figure 5). *Gloeophyllum trabeum* had no detectable expression of FIBRA_01430 on untreated and copper-treated wood over the course of this study thus no data are shown. Unlike FIBRA_00974 and FIBRA_04716 expression patterns, both *F. radiculosa* isolates (FP-90848-T and L-9414-SP) upregulated FIBRA_01430 on untreated and copper-treated wood (*p* = 0.481 and *p* = 0.392, respectively). However, *F. radiculosa* FP-90848-T (Figure 5A) increased in FIBRA_01430 upregulation on 0.6 and 2.4% copper-treated wood over time (*p* = 0.000).

## Discussion

To extend the service life of treated-wood products, it is critical to determine the mechanisms by which decay fungi can deteriorate wood. Due to the Environmental Protection Agency’s restriction of chromated copper arsenate (CCA) in 2004, arsenic-free and chromium-free preservatives utilize copper as the primary means of protection against biological organisms (Lebow, 2004, 2010; Lebow et al., 2004; Freeman and McIntyre, 2008). Copper-tolerant brown-rot fungi are quite problematic because of their ability to degrade copper-treated wood products in service. Copper tolerance necessitates the typical inclusion of co-biocides in commercial preservatives treatments (Illman et al., 2000). But to better understand how F. radiculosa responds to copper without co-biocides, we used the model copper preservative, copper citrate (Clausen et al., 2000; Clausen and Green, 2003; Hastrup et al., 2005), for this study. In addition, we were interested in how *F. radiculosa* initiates decay; therefore, we chose to determine mass loss during early decay (weeks 1 through 4).

Mass loss results of this study are consistent with previous studies which determined that *G. trabeum* is significantly inhibited by copper-treated wood (Green and Clausen, 2003; Hastrup et al., 2005, 2006; Jenkins et al., 2014; Ohno et al., 2017). Humar et al. (2002) treated Norway spruce (*Picea abies*) with copper sulfate and copper octanoate with ethanolamine (CuE) and showed *G. trabeum* inhibition on both copper-treatments by 4 weeks (3.9 and 0.0% mass loss, respectively). Green and Clausen (2003) reported 0% mass loss of 1.2% CC-treated wood by *G. trabeum* at 10 weeks. Humar et al. (2006) found *G. trabeum* inhibition on Norway spruce treated with copper sulfate with potassium dichromate (CuCr) and copper sulfate with CuE at weeks 1, 2, and 4 with only 0.3% mass loss on CuCr and 0.4% mass loss on CuE by week 4. Hastrup et al. (2006) treated SP with 1.2% CC and showed 4.8% mass loss by *G. trabeum* at week 10. Liew and Schilling (2012) reported similar effects of copper CuE-treated and micronized copper quaternary-treated wood on *G. trabeum* by week 12 (4.9 and 2.1% mass loss, respectively). Jenkins et al. (2014) also found copper sensitivity of *G. trabeum* on 1.2% CC-treated wood at weeks 2 and 4; however, there was no copper sensitivity when SP was not pressure-treated with copper (i.e., dipped in copper, adjacent to copper). In each of these studies, the copper effects on decay by *G. trabeum* were similar to those of the present study.

Mass loss results of this study are also consistent with previous studies of decay of copper-treated wood by *F. radiculosa*. Clausen and Green (2003) reported similar mass loss by *F. radiculosa* (formerly *Meruliporia incrassata*) TFFH 294 on CC-treated wood for weeks 1 through 4 (1.6, 3.5, 3.6, and 8%, respectively). Results of Green and Clausen (2003) also showed high mass loss by *F. radiculosa* (formerly *Antrodia radiculosa*) FP-90848-T and *F. radiculosa* TFFH 294 on 1.2% CC-treated wood at 10 weeks (49 and 45%, respectively). They also reported mass loss to be greater on copper-treated wood compared to untreated wood for FP-90848-T and TFFH 294 (26 and 37%, respectively) at 10 weeks (Green and Clausen, 2003).

Among *F. radiculosa* isolates, there is diversity in timing and levels of gene expression. Differences in *F. radiculosa* isolates seen in this study were consistent with previous work suggesting *F. radiculosa* L-9414-SP has a delayed response in adapting to copper when compared to other *F. radiculosa* isolates (Jenkins, 2012; Ohno et al., 2015). Additional isolates of *F. radiculosa* (TFFH 294 and L-11659-SP) have been documented to have differences in oxalate accumulation and differential expression of genes encoding a citrate synthase, an isocitrate lyase, a glyoxylate dehydrogenase, a succinate/fumarate antiporter, and a copper transporting ATPase pump over the course of 8 weeks (Ohno et al., 2015). Zelinka et al. (2018) used synchrotron-based X-ray fluorescence microscopy to observe copper distribution in SP wood wafers that were dipped in copper sulfate pentahydrate after exposure to three copper-tolerant fungi, *F. radiculosa*, *P. placenta*, and *F. (Tyromyces) palustris*. After exposure to *F. radiculosa* for 9 weeks, there were much lower copper concentrations remaining in the dipped wafers, but only slightly lower copper concentrations after *P. placenta* and *F. palustris* exposure. They suggested that copper-tolerant species could rely on unique undefined processes for copper-tolerance. It has also been documented that brown-rot fungi in general have variations in the biochemical processes of decay and are highly dependent upon the environment in which they are grown (Presley and Schilling, 2017). From these studies mentioned above, it could be inferred that the necessary components in copper-tolerance are unique to a particular species and even isolate.

A possible mechanism of copper-tolerance is translocation of copper out of the area of fungal growth. Previous authors have reported that preservative tolerant fungi appear to be able to mobilize preservative elements, including copper, and decrease their concentration in the affected zone (Choi et al., 2012; Hastrup et al., 2014; Zelinka et al., 2018). Relatively few studies have investigated copper-tolerant *F. radiculosa* for other intracellular methods to overcome the toxic effects of copper (Tang et al., 2013; Ohno et al., 2015). Tang et al. (2013) reported minimal upregulation of one ATPase gene (FIBRA_01430) at week 3 when *F. radiculosa* was grown on wood treated with micronized copper quaternary. In addition, Tang et al. (2013) found no correlation between ATPase expression and mass loss. Ohno et al. (2015) reported upregulation of one ATPase gene (FIBRA_01430) at weeks 2 and 4 when *F. radiculosa* was grown on copper citrate-treated wood. From that study, Ohno et al. (2015) hypothesized that the mechanism of copper-tolerance is dependent on the intracellular management of copper facilitated by the copper-transporting ATPase genes in combination with extracellular oxalic acid accumulation and precipitation in *F. radiculosa*.

ATPase gene expression in other filamentous fungi is limited. *Rhizophagus irregularis*, an arbuscular mycorrhizal fungus, can tolerate high concentrations of metals in soils as a result of antioxidant defense activation. Tamayo et al. (2014) analyzed four ATPase encoded genes in *R. irregularis*, three characterized ATPase genes comparable to *S. cerevisiae* ATPases (i.e., shuttles copper ions to copper dependent enzymes) and one characterized as a plasma membrane ATPase comparable to *C. albicans* (i.e., copper detoxification). They showed upregulation of the three *S. cerevisiae*-like ATPases indicating a role in supplying copper to enzymes required for fungal establishment; however, they showed no expression data on the one *C. albicans*-like ATPase. In the corn pathogen, *Cochliobus heterostrophus*, Saitoh et al. (2009) analyzed heavy metal ATPase genes, which were divided into three groups. Group A, which delivers copper to copper-dependent proteins, and groups B and C, which are fungal specific and function in cell membrane copper efflux (Saitoh et al., 2009). However, this was just a phylogenetic analysis and no expression data were shown for the three groups characterized. Uldschmid et al. (2003) identified an ATPase protein, *ctaA*, in the white-rot fungus, *Trametes versicolor*. They showed *ctaA* was upregulated in the presence of copper and downregulated in the absence of copper and concluded this ATPase is localized to the Golgi membrane and functions in providing copper to copper-dependent enzymes, like laccase (Uldschmid et al., 2003).

The current study focused on analyzing intracellular ATPase genes to determine if they are necessary in the mechanism of copper-tolerance in *F. radiculsoa*. Copper-sensitive *G. trabeum* had greater FIBRA_00974 upregulation when grown on all copper-treatments (Figure 3A). In addition, *G. trabeum* also exhibited greater FIBRA_04716 upregulation when grown on all copper-treatments at week 1 (Figure 4A). These results suggest *G. trabeum* was attempting to manage the unfavorable copper environment, but ultimately was not successful, indicated by the lack of mass loss in copper-treated wood (Figure 1A).

The *F. radiculosa* isolates downregulated FIBRA_00974 (Figures 3B,C) and FIBRA_04716 (Figures 4B,C) after week 1, but upregulated FIBRA_01430 (Figures 5A,B). Additionally, both isolates showed no significant upregulation in expression of FIBRA_00974, FIBRA0416, and FIBRA_01430 after week 1 on copper-treated wood. These results suggest *F. radiculosa* does not rely upon intracellular copper management when exposed to increasing concentrations of copper. *F. radiculosa* still managed to cause decay on copper-treated wood (Figures 1B,C); however, this is likely not facilitated by the copper-transporting ATPase gene in *F. radiculosa*.

*Gloeophyllum trabeum* and *F. radiculosa* L-9414-SP had similar FIBRA_00974 (Figures 3A,C) and FIBRA_04716 (Figures 4A,C) upregulation on copper-treated wood at week 1. *Gloeophyllum trabeum* continued this trend in FIBRA_00974 upregulation at weeks 2 and 3 on copper-treated wood. *Fibroporia radiculosa* L-9414-SP also increased FIBRA_00974 and FIBRA_04716 upregulation with increasing copper-concentration. However, by week 2, there were no significant differences in FIBRA-00974 and FIBRA_0416 expression on untreated and copper-treated wood by this *F. radiculosa* isolate. This result is interesting and could suggest *F. radiculosa* L-9414-SP, like *G. trabeum*, responded similarly to an unfavorable copper environment at week 1. These results might suggest that FIBRA_00974 (and possibly FIBRA_04716) are involved in a mechanism of copper susceptibility in decay fungi; however, additional research is needed to address this hypothesis.

Expression of three copper-transporting ATPase genes thought to be involved in the mechanism of copper tolerance was quantified for the first time. Additionally, ATPase expression between copper-sensitive and copper-tolerant decay fungi during early decay of untreated and copper-treated wood had not been compared previously. This was one of a few studies in which intracellular methods for overcoming toxic copper concentrations in decay fungi were evaluated. *Gloeophyllum trabeum* had greater FIBRA_00974 and FIBRA_04716 upregulation when grown on copper-treated wood compared to untreated wood; however, this fungus was not successful at decay of copper-treated wood. *Fibroporia radiculosa* had similar ATPase expression on untreated and copper-treated wood (with the exception of FIBRA_00974 and FIBRA_04716 at week 1), and thus showed the same capacity to decay copper-treated wood as untreated wood (Figures 1B,C).

Environmental stresses such as metal toxicity (i.e., copper toxicity) enhance ROS production and threaten cell viability by causing a variety of issues (e.g., lipid peroxidation, protein oxidation, nucleic acid damage, enzyme inhibition, and programmed cell death; Sharma et al., 2012). To avoid oxidative injury, cells control ROS levels by means of non-enzymatic and enzymatic antioxidants. Enzymatic antioxidants include a wide variety of enzymes including superoxide dismutases, catalases, and peroxidases and increased activity of these enzymes deal with environmentally induced oxidative stress (Noctor and Foyer, 1998; Sharma et al., 2012). Increased metal tolerance has been linked to antioxidant capacity to scavenge toxic ROS and simultaneous expression of multiple antioxidant enzymes is more effective (Lee et al., 2007; Zaefyzadeh et al., 2009; Chen et al., 2010). In fact, it has been suggested that specific cellular responses to unfavorable environments are induced in decay fungi. These responses are: (a) production of laccases, peroxidases, and other lignin modifying enzymes (Peralta et al., 2017); (b) tolerance of oxidative stress and high levels of ROS facilitated by high levels of antioxidant enzymes like superoxide dismutase, catalase, and glutathione peroxidase an glutathione reductase (Belinky et al., 2003; Jaszek et al., 2006; Maciel et al., 2013); and (c) production of intracellular cytochrome p450 monooxygenases (Magan et al., 2010; Coelho-Moreira et al., 2013, 2018). It is highly possible that one or even several of these antioxidant enzymes could be implicated as critical components in the mechanism of copper-tolerance in decay fungi; however, additional research is needed to verify this statement. The mechanism of copper-tolerance in decay fungi remains unclear. Oxalic acid has been implicated in extracellular management of copper by decay fungi but intracellular management of copper by decay fungi remains unknown. Results of this research suggest copper-tolerant fungi like *F. radiculosa* utilizes a different mechanism to decay copper-treated wood when compared to copper-sensitive fungi like *G. trabeum*. Roles of the three copper-transporting ATPases in overcoming toxic copper environments in copper-tolerant fungi could not be confirmed, but differences were observed in gene expression between fungi and between untreated and copper-treated wood. Although this study did not pinpoint critical components of intracellular copper management by copper-tolerant brown-rot decay fungi, future studies can use the information provided to determine a detailed explanation of the mechanism of copper tolerance. Understanding this mechanism will prove critical in reducing the destruction of treated-wood products by copper-tolerant brown-rot fungi. Ultimately, additional research is needed to determine how *F. radiculosa* manages copper intracellularly. Additional studies should perhaps focus on analyzing gene expression without the wood substrate (i.e., liquid media) to better understand how certain interactions are happening. In addition, there are a multitude of stress-response genes (i.e., laccases, catalases, superoxide dismutases, cytochrome p450 monooxygenases, thioredoxins, etc.) that could play a role in copper-tolerance in wood decay fungi and should be investigated. Future research to comprehensively explain decay initiation by copper-tolerant brown-rot decay fungi could eventually lead to knowledge of how copper-tolerant brown-rot decay fungi manage toxic copper environments.

## Data Availability Statement

The raw data supporting the conclusions of this article will be made available by the authors, without undue reservation.

## Author Contributions

AB contributed technical and laboratory assistance, and aided in the materials and methods write up. All authors contributed to the article and approved the submitted version.

### Conflict of Interest

The authors declare that the research was conducted in the absence of any commercial or financial relationships that could be construed as a potential conflict of interest.
